# Bilateral Recurrent Uveitis in a Young Patient with Family History of Spondyloarthritis: Spondyloarthritis or Not?

**DOI:** 10.31138/mjr.32.3.273

**Published:** 2021-09-30

**Authors:** Nikolaos Kougkas, Nestor Avgoustidis, Eleftheria-Kleio Dermitzaki, Harikleia Gakiopoulou, Kostas Stylianou, George Bertsias

**Affiliations:** 1 Department of Rheumatology, Clinical Immunology and Allergy, University of Crete School of Medicine, Heraklion, Greece; 2 Nephrology, Heraklion University Hospital, Heraklion, Greece; 3 Nephropathology, National and Kapodistrian University of Athens, Athens, Greece

**Keywords:** uveitis, tubulointerstitial nephritis, spondyloarthritis, TINU

## Abstract

We present the case of a young man with a strong family history of SpA, who was referred to the Rheumatology Clinic due to bilateral uveitis refractory to treatment with corticosteroids. The patient’s renal function gradually deteriorated and a subsequent biopsy was positive for interstitial nephritis. After excluding all other systemic diseases, the diagnosis of TINU syndrome was confirmed. Although rare, TINU syndrome should be considered in the differential diagnosis of non-infective uveitis especially in the presence of urinalysis abnormalities.

## INTRODUCTION

Non-infectious uveitis is an intraocular, sight-threatening inflammation which is divided as anterior, intermediate, posterior or panuveitis according to the affected equator of the eye.^1^ It is a relatively common disease which can be either idiopathic or more often associated to underlying systemic autoimmune diseases. In the latter case, uveitis can be the presenting manifestations, thus making diagnosis a challenging task.

An 18-year-old patient with family history of spondyloarthritis (SpA) (first degree relative with Ankylosing Spondylitis and Crohn’s Disease) was referred from the ophthalmologist due to new-onset bilateral anterior uveitis. Work-up had excluded infectious diseases and the patient had already received topical and oral corticosteroids with modest improvement.

From the clinical examination, there were no findings of SpA or any other systemic autoimmune disease.

Chest X-ray was normal, HLA-B27 was negative and the labs were only remarkable for raised serum creatinine of 1.4 mg/dL (glomerular filtration rate of 84.7 ml/min) from 0.9 mg/dl in the beginning of the clinical syndrome.

The patient had not received any potential nephrotoxic drugs.

To expedite the tapering of corticosteroids, methotrexate (MTX) was started at a dose of 15 mg/week with 5 mg/week folic acid supplementation. A month later, serum creatinine remained increased at 1.5mg/dl, while all other labs were normal. At that time urine sediment revealed the presence of epithelial and white blood cell casts, while the kidney ultrasound was normal and autoantibodies ANA, ENA and ANCA were negative.

**Figure 1.**
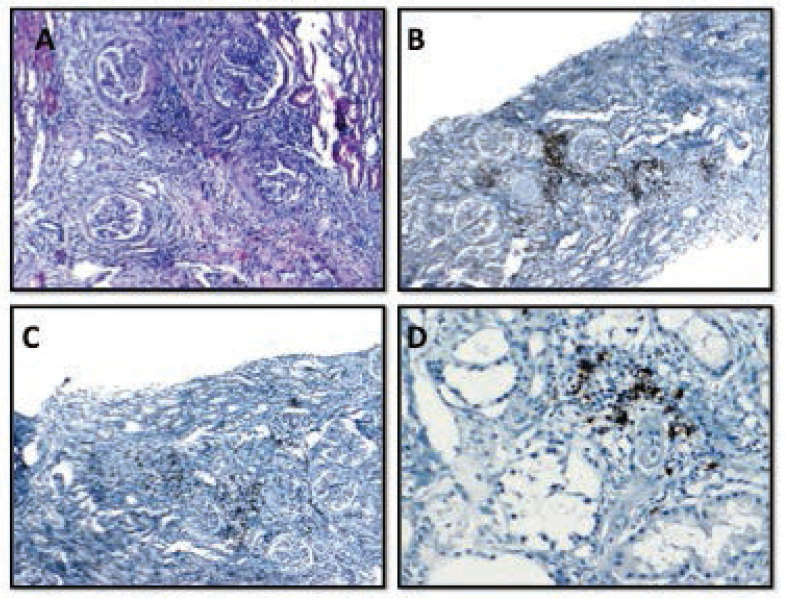
(A) Moderate interstitial infiltration of the kidney by inflammatory cells, Hematoxylin-Eosin x100 (B) Immunohistochemistry for CD4 positive cells, x100. T CD4 lymphocytes were the predominant inflammatory cell population infiltrating the kidneys. (C) Immunohistochemistry for CD8 positive cells, x100 (D) Immunohistochemistry for CD20 positive cells, x400

Because of the persistent – albeit modest – impairment of renal function, a kidney biopsy was performed in order to rule out any systemic disease affecting both organs (eyes and kidneys). Indeed, kidney biopsy revealed interstitial nephritis (affecting 20% of the cortex) with predominant B- and T-lymphocytic infiltration and only rare plasma cell and macrophage infiltrates (**Figure 1**); 2 out of 63 glomeruli were globally sclerotic with the remainder being normal, and there were no significant immune deposits by immunofluorescence or electron microscopy. As tuberculosis and sarcoidosis are commonly associated with interstitial nephritis and uveitis, tuberculosis (TB) interferon-gamma release assay (IGRA) in the blood and serum angiotensin converting enzyme levels were performed and were negative.

Based on the biopsy findings and after the exclusion of all other possible diagnoses from the clinical and laboratory work up, the patient was diagnosed with Tubulointerstitial nephritis and uveitis (TINU) syndrome. He received pulses of intravenous methylprednisolone and continued with methotrexate as a steroid-sparing agent. Although uveitis fully responded to treatment, serum creatinine levels remained stable and the patient developed mild arterial hypertension (140/90 mmHg) that required treatment with angiotensin converting enzyme (ACE) inhibitors. A new course of corticosteroids was administrated and MTX was switched to azathioprine (AZA), resulting in an improvement of renal function with a sustained alleviation of uveitis.

## DISCUSSION

TINU syndrome is a rare autoimmune disorder affecting mainly adolescents and young adults. It is characterised by the combination of uveitis (usually anterior bilateral) and tubulointerstitial nephritis.^2^

The diagnosis of the syndrome is based on the clinical manifestations, the consistent renal biopsy, and the exclusion of other diseases, including systemic infections (eg, tuberculosis) and autoimmune diseases such as ANCA-associated vasculitis, systemic lupus erythematosus, Sjögren’s syndrome and sarcoidosis.

The prognosis is usually favourable, although cases of severe and persistent disease have been described. Due to its rarity, there are no evidence-based therapeutic protocols. The mainstay of treatment is corticosteroids and in refractory cases, immunosuppressive agents such as azathioprine, methotrexate, cyclophosphamide and mycophenolate mofetil have been used.^3^

Uveitis is an inflammatory condition frequently referred to rheumatologists, especially when it is recurrent, with the suspicion of a systemic autoimmune disorder (**Table 1**). Indeed, in many cases, and especially in SpA, it can be the presenting symptom of the disease.^4^ In most cases, inflammation is localised in the anterior chamber of the eye (anterior uveitis, also called iritis), but can also found in the vitreous and in retina and choroid (intermediate and posterior uveitis respectively). In our case, the patient was referred with this possible diagnosis due to his young age and family history for SpA. Notwithstanding, the absence of supporting clinical, laboratory and imaging findings, in combination with the gradual renal function impairment, pointed towards another diagnosis. Therefore, in patients who present with the combination of uveitis and renal involvement (impaired renal function and/or cellular casts), alternative diagnoses such as the TINU syndrome should be considered.

**Table 1.** Uveitis related to autoimmune diseases.

Spondyloarthritis
Inflammatory Bowel Disease
Juvenile idiopathic arthritis
Behçet’s Disease
Sarcoidosis
Vogt-Koyanagi-Harada Syndrome
Vasculitis
Kawasaki’s Disease
Blau Syndrome
Neonatal onset multisystem inflammatory disease
Interstitial nephritis (TINU syndrome)
Multiple sclerosis
Relapsing polychondritis
Sjögren’s Syndrome
Systemic lupus erythematosus
Vitiligo
Sweet syndrome
